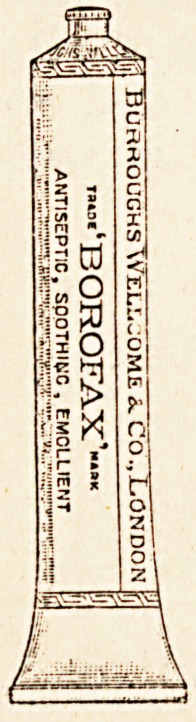# Notes on Preparations for the Sick

**Published:** 1905-06

**Authors:** 


### Notes on Preparations for the Sick.

[For the analyses given in these notes we are indebted to Mr. Oliver C. M. Davis, B.Sc., of the University College Laboratory.—Ed.]

**Digitaline Crystallised: Nativelle's granules.** — Wilcox, Jozeau & Co., London.—This preparation of Digitalis has long been in use, but it is becoming more and more recognised that a standard preparation of absolutely uniform composition presents advantages as a substitute for the crude Digitalis and its various preparations. Each granule contains exactly a quarter of a milligramme, or $\frac{1}{240}$th of a grain. The dose is given as one to four granules in twenty-four hours. This must be taken as the maximum dose ($\frac{1}{60}$th grain in twenty-four hours). We have usually found that one granule daily for five or six days will not fail to demonstrate the physiological results of Digitalis. They are much too active to be entrusted to patients who are not under medical supervision, and it is well to suspend the daily use from time to time.

**Tabloids.**—Burroughs, Wellcome & Co., London.

**Quinine, Camphor and Aconite.**

| | |
|---|---|
| Quin. Sulph. ... ... ... ... ... ... | gr. $\frac{1}{4}$ |
| Camphoræ ... ... ... ... ... ... | gr. $\frac{1}{4}$ |
| Tinct. Aconiti ... ... ... ... ... ... | ♏j |

A convenient and valuable diaphoretic in catarrhal conditions.

**Ferric Chloride and Arsenic.**

| | | |
|---|---|---|
| Tinct. Ferric Chloride | ... ... | ♏x |
| Arsenious Acid | .. ... ... ... | gr. $\frac{1}{30}$ |

The hæmatinic value of iron is often enhanced by the addition of arsenic, which is found to increase the number of red blood cells in anæmic conditions, whilst iron increases chiefly the amount of hæmoglobin in the corpuscle. Arsenic is also a valuable nerve tonic. This preparation has a special value in pernicious anæmia, in leucocythæmia and in nervous diseases. The method of administration may vary. Usually one tabloid is dissolved in a large wineglassful of water and swallowed after food. "Tabloid" Ferric Chloride and Arsenic should never be taken undissolved on an empty stomach. It may be swallowed whole, however, if taken with or immediately after food, with a large draught of water.

**Hemisine: Tabloid Hemisine,** gramme 0.0003; **Tabloid Ophthalmic** (**CC**) **Hemisine,** gramme 0.0006; **Soloid Hemisine,** gramme 0.005; **Soloid Hemisine,** gramme 0.0012; **Enule Hemisine,** gramme 0.001.—Soloids gr. $\frac{1}{12}$ and Ophthalmic Tabloids gr. $\frac{1}{100}$ provide a convenient and reliable form of suprarenal gland extract. The tabloids keep well and are easily soluble. We have used this drug freely in operations on the conjunctiva and ocular muscles and found it act very satisfactorily. With its assistance such operations as advancement of the recti muscles are much simplified owing to the almost complete absence of hemorrhage. A fresh $\frac{1}{1000}$ solution is recommended, but a much weaker solution acts almost as well, and will retain its activity for several days. Therapeutically it is useful in many forms of conjunctival hyperæmia, but in deep-seated inflammation of the eyeball its application is of doubtful benefit. Hemisine combined with eucaine would appear to be an almost ideal spray for nasal operations.

**Borofax.**—Borofax is an emollient possessing antiseptic and sedative properties. It is superior to ointment or glycerine of boric acid in therapeutic action, readiness of absorption, pharmaceutical elegance, and freedom from rancidity. It encourages healing of superficial lesions, and therefore is a valuable application for chaps, burns, scalds, and abrasions.

Borofax may be used with advantage in the treatment of eczema and many other skin affections, and also in excoriation or disease of mucous surfaces.

It is beneficial and soothing to the skin after driving, motoring, cycling, or exposure to extremes of temperature.

Borofax is supplied in collapsible tubes of two sizes. It is pleasantly perfumed, and of convenient consistence for application to delicate surfaces.

**Gadd's Standardised Galenicals: Ext. Ergot. Liq.; Tinct. Aconiti; Tinct. Cannab. Ind.; Tinct. Digitalis; Tinct. Scillæ; Tinct. Strophanthi.**—EVANS, GADD & Co., Bristol and Exeter.—These tinctures have all been prepared to meet the requirements of the British pharmacopœia, and in addition their activity has been determined by the application of physiological tests.

In the numerous cases in which the value of a preparation cannot be satisfactorily determined by chemical examination, it is desirable that their physiological activity should be tested for the satisfaction of the physician as well as the benefit of the patient.

---

**Xeroform.**—BURGOYNE, BURBIDGES & Co., London, E.C.—This substitute for iodoform, from the Chemische Fabrik von Heyden, is practically odourless, tasteless, not poisonous, and not irritating. It contains 50 per cent. of bismuth oxide in chemical combination with 50 per cent. of tribromphenol: it is therefore chemically tribromphenol-bismuth. It is a fine, yellow powder of neutral reaction, with a slight phenol-like odour, almost tasteless, and unaffected by light. It is insoluble in water, alcohol, vegetable oils, fluid vaseline and chloroform. It is soluble in 2 per cent. hydrochloric acid in the proportion of 30.100. With alkalies it forms bromides. It is decomposed by a temperature of over 120° C. (248° F.) only. This is of importance, since it shows that the drug can be readily sterilised.

The bromine derivative of phenol has been rendered suitable for internal and external use by the introduction of the bismuth with the molecule. It is intended for use as an intestinal disinfectant and for surgical purposes. From the chemical point of view there is every reason to expect that it will justify the makers' claims and become well known as an improvement on the objectionable iodoform, which it closely resembles in appearance.

---

**Ferroleum, the Iron-oil-food.**—THE FERROLEUM Co., 86 Clerkenwell Road, London, E.C.—An emulsion of cod liver oil with iron and phosphorus, with the following formula:—

| | |
|---|---|
| Olei Morrhuæ ... ... ... ... ... ... | ℥vij |
| Ferri Phosphat... ... ... ... ... ... | ʒij |
| Phosphori ... ... ... ... ... ... ... | gr. i |
| Glycerini, &c., q.s. ad ... ... ... ... | ℥xv |

It appears to be a perfect and a durable emulsion, in which the *Lancet* analysis shows the presence and amount of constituents as described.

Our examination reveals the presence of a good percentage of oil in combination with emulsifying agents and some phosphate of iron. The microscope shows it to be a good and uniform emulsion which does not tend to separate. It is faintly acid, the reaction being due to traces of free phosphoric acid, which holds the iron in solution. Being pleasant to the taste, owing to the presence of flavouring agents, it should be acceptable to children and the fastidious adult, whilst its physical properties are such that it should be readily digested and absorbed.

---

**Fructol. Medullæ et Glycerophos. (Fructole of Red Bone Marrow and Glycerophosphates)**, a new chemical food.—SAVORY & MOORE, London.—This preparation has a fruit basis in which all the sugar is replaced by purified glycerine. One ounce contains the equivalent of

| | |
|---|---|
| Red Bone Marrow ... ... .. ... | ʒj |
| Iron Glycerophosphate ... ... | gr. ij |
| Calcium ... ... ... ... ... | gr. iv |

One to four teaspoonfuls may be taken with water three times a day after food.

Other Fructole combinations are made by this firm, the most important of which are the Fructole of the Glycerophosphates, Fructole of the Hypophosphites, and Fructole of Terpin and Heroin.

---

**Tamar Indien Grillon.**—GUÉNIN & Co., Paris.—We frequently meet with this well-known laxative; it has been in use for thirty years, and has not yet been displaced by the numerous more recent preparations of a laxative character. We are not allowed to know anything of its composition, but experience has shown that it is not harmful, and that it is what it professes to be—a safe and simple laxative in an agreeable form.

---

**Cognet's Dragées.**—43 Rue de Saintonge, Paris.—These are a compound of protoxalate of iron and crystallised quassine. They have had a long experience as an active hæmatinic: they are easily assimilated and do not commonly give rise to gastric pains or constipation. Two or three may be taken twice daily with meals.

---

**Neurisine** (Prunier), a granulated form of pure Glycerophosphate of Calcium.—CHASSAING & Co., Paris (64 Holborn Viaduct, London).—It is also prepared in the form of syrup and in wafer cachets. The constant presence of phosphorus in the nerve tissues in the form of lecithine is supposed to demonstrate its importance in the production of vital phenomena. The glycerophosphoric acid contained in the lecithines is believed to be the most essential element of the nucleins which are extracted from cell nuclei. The elimination of phosphorus from the body is easy and unceasing, the mineral compounds of phosphorus are difficult of assimilation, and therefore do not supply the waste. Hence the organic compounds have become quite fashionable in all conditions where an excessive loss of phosphates is believed to be associated with nervous waste. The glycero-phosphates are much used in a variety of neurasthenic conditions: the phosphorus exists in a readily-assimilable form, and good results should be expected from their continued use.

---

**The Lock and Key Bottle.**—Thos. Christy & Co., London.—In consequence of the occasional accidental poisoning by carbolic acid and other concentrated poisons, and in spite of the care which is usually exercised to dispense such fluids in special bottles of various kinds, it has become necessary to go one step further and provide a lock and key. The patent locked bottle containing the usual quality of carbolic acid used for disinfecting purposes is supplied by Messrs. Thomas Christy & Co. at the same price as commonly sold in any ordinary bottle. This firm also supplies empty poison bottles with a nickel-plated patent automatic lock and key for locking up all poisons. These bottles will, of course, be very useful if steps be taken to insist on their use, otherwise the little trouble they involve will soon lead to their neglect.

## Figures and Tables

**Figure f1:**